# Draft Genome Sequence of *Ustilago trichophora* RK089, a Promising Malic Acid Producer

**DOI:** 10.1128/genomeA.00749-16

**Published:** 2016-07-28

**Authors:** Thiemo Zambanini, Joerg M. Buescher, Guido Meurer, Nick Wierckx, Lars M. Blank

**Affiliations:** a Institute of Applied Microbiology (iAMB), Aachen Biology and Biotechnology (ABBt), RWTH Aachen University, Aachen, Germany; b BRAIN AG, Zwingenberg, Germany

## Abstract

The basidiomycetous smut fungus *Ustilago trichophora* RK089 produces malate from glycerol. *De novo* genome sequencing revealed a 20.7-Mbp genome (301 gap-closed contigs, 246 scaffolds). A comparison to the genome of *Ustilago maydis* 521 revealed all essential genes for malate production from glycerol contributing to metabolic engineering for improving malate production.

## GENOME ANNOUNCEMENT

The members of the family *Ustilaginaceae*, belonging to the phylum *Basidiomycota*, are known to naturally produce many different industrially interesting compounds, such as organic acids, lipids, and polyols (1–6). In a screening of 74 strains belonging to 13 species, *Ustilago trichophora* RK089 (CBS 131473) showed the highest malic acid production from glycerol (7). After adaptive laboratory evolution and medium and process optimization, this strain was capable of producing more than 200 g liter^-1^ malic acid at a maximum production rate of nearly 2 g liter^-1^ h^-1^, demonstrating its potential as a production organism (8). *U. trichophora* was first isolated from *Echinochloa colonum* in Egypt by Kunze in 1830 (9). Since then, this organism has attracted little focus of research, apart from the description as a plant pathogen belonging to the *Ustilaginaceae*, including its phylogeny (10–13). Hence, no prior knowledge exists about molecular techniques for genetic manipulation, and the genome sequence is unknown. For further optimization of malate production by *U. trichophora*, however, metabolic engineering is required.

Here, we present a draft genome sequence of *U. trichophora* RK089. Sequencing and *de novo* assembly were done by BaseClear BV (Leiden, The Netherlands) using Illumina Nextera (paired-end library) and PacBio RSII (10-kb library) with a single-molecule real-time (SMRT) cell for sequencing. CLC Genomics Workbench version 7 was used for draft assembly of the reads using the “De novo assembly” option. The optimal k-mer size was automatically determined using KmerGenie (14). Alignment of the Pac Bio continuous long reads (CLR) was performed with BLASR (15). Analysis of the orientation, order, and distance between resulting contigs was performed using SSPACE‑LongRead scaffolder version 1.0 (16), and automated gap closure was performed using GapFiller version 1.10 (17). The resulting sequence of 246 gap-closed scaffolds contains 20,691,595 bp consisting of 1,399 large contigs (>300 bp in size) and 77 smaller contigs, with a G+C content of 53.06% and 124 gaps. The average sequence size is 84,112 bp, with a maximum of 637,988 bp.

In a comparison of the sequence with the closely related *Ustilago maydis*, several genes expected to be involved in the conversion of glycerol to malic acid were identified that have 88 to 90% homology at the DNA level and 94 to 97% homology at the protein level. None of the genes from the itaconic acid cluster, which were recently discovered for *U. maydis* (18), are present in *U. trichophora* RK089, a strain that indeed does not produce itaconic acid. We were able to identify all genes from *U. maydis* glycolipid clusters, i.e., those coding for ustilagic acid (UA) (19) and mannosylerythritol lipid (MEL) (20) synthesis.

### Nucleotide sequence accession numbers.

This whole-genome shotgun project has been deposited in DDBJ/ENA/GenBank under the accession no. LVYE00000000. The version described in this paper is version LVYE01000000.
